# 

**Published:** 1886-07

**Authors:** 


Page.

ORIGINAL COMMUNICATIONS:

The Relationship of the Bacterium Tuberculosis to the Tubercles of the Human Lungs and Other Organs. H. D. Schmidt ........ 579

Agorophobia. A. K. Van Horn .... 600

EDITORIAL:

Actinomycosis in Man ........ 604

The Daily Press and Medical Societies ........ 605

Professor v. Gudden ........ 606

SOCIETY REPORTS:

CHICAGO MEDICAL SOCIETY. *Meeting May 3rd.*

Double Hare-Lip and Cleft-Palate in an Infant. M. P. Kossakowski 606

Pure Drinking-Water. H. Gradle. 606

Scleroderma. W. H. Lyford ...... 609

Undiagnosable Maladies. J. I. Tucker ........ 613

Anchylosotomum Duodenale. W. T. Belfield ........ 615

Tenia Mediocaellata. W. W. Jaggard ........ 616

Michigan State Medical Society .... 616

Page.

TRANSACTIONS OF THE CHICAGO GYNÆCOLOGICAL SOCIETY. *Meeting April 23rd.*

Hernia of the Umbilical Cord. C. Caldwell ........ 618

Dermoid Cyst, Complicating Labour. John Bartlett ........ 629

Placenta Prævia. John Bartlett ... 630

FOREIGN CORRESPONDENCE:

The Italian Surgical Society. A. Lagorio ........ 641

Professor Credé's Clinic in Leipsic. W. S. Caldwell ........ 648

DOMESTIC CORRESPONDENCE:

Notes of Medico-Legal Cases. S. V. Clevenger ........ 656

"Insane Hospital Supervision," and The State Board of Public Charities of Illinois. F. H. Wines ........ 663

BOOK REVIEWS:

Insanity and its Treatment. G. F. Blandford ........ 669

A Treatise on Nervous Diseases. S. G. Webber ........ 670

Diseases of the Spinal Cord. B. Bramwell ........ 671

Treatise on Nasal Catarrh and Allied Diseases. B. Robinson ..... 672

